# Influence of the COVID-19 Pandemic on Quality of Life of Patients with Parkinson's Disease

**DOI:** 10.1155/2020/1216568

**Published:** 2020-10-05

**Authors:** Dengjun Guo, Bing Han, Yuqiang Lu, Chenling Lv, Xiaoling Fang, Zhenzhong Zhang, Zhenguo Liu, Xiaoping Wang

**Affiliations:** ^1^Tongde Hospital of Zhejiang Province, Hangzhou, Zhejiang 310012, China; ^2^Xinhua Hospital, Shanghai Jiaotong University Medical School, Shanghai 200092, China; ^3^Tongren Hospital, Shanghai Jiao Tong University Medical School, Shanghai 200336, China

## Abstract

**Introduction:**

This study investigated the influence of lockdown during the 2019 coronavirus disease (COVID-19) pandemic on the quality of life of patients with Parkinson's disease (PD).

**Methods:**

We conducted a questionnaire survey involving 113 patients with PD from Xihu District, Hangzhou, Zhejiang. During the epidemic prevention and control period (February 1 to March 31, 2020), patients enrolled were asked to fill out questionnaires, including the “COVID-19 Questionnaire for PD Patients during the Period of Epidemic Prevention and Control” and “39-item Parkinson's Disease Questionnaire (PDQ-39).” During the phase of gradual release of epidemic prevention and control (April 1 to April 30, 2020), all patients were followed up again, and PDQ-39 questionnaires were completed.

**Results:**

The quality of life for patients during the period of epidemic prevention and control was worse than that after epidemic prevention and control (*P* < 0.001). The biggest problem that they faced was that they could not receive their doctor's advice or guidance regularly. The quality of life of patients who had difficulty getting doctors' guidance or those who changed their routine medication due to lockdown was even worse. Telemedicine was quite effective and efficient for patients to get doctors' guidance during lockdown.

**Conclusions:**

The inconvenient treatment during the pandemic directly caused the aggravation of patients' symptoms and the decline in their quality of life. It is suggested that social media (such as WeChat or Tencent QQ) are used for regular interactions and follow-up appointments for patients with inconvenient medical treatment.

## 1. Introduction

In the past several months, the coronavirus disease (COVID-19) outbreak has largely changed the rhythm of human life and overwhelmed the healthcare systems of many countries [1]. Many provinces in China, including Zhejiang, Guangdong, and Hunan, successively launched first-class responses to major public health emergencies, after lockdown measures were imposed in Wuhan, Hubei Province, at 10 a.m. on January 23, 2020. Since then, especially between February and March 2020, people were asked to stay at home as much as possible and not travel outside without specific reasons. Each family was given two passes per week to go out to purchase food and other necessities. Each pass could only be used by one person. Furthermore, a health green code was required to be shown when people entered public places. The travel restriction order was enforced by the government aimed at preventing the spread of the virus and protecting people from infection. However, it also led to the stagnation of social and economic life. Hospitals began reducing outpatient services and limiting daily visits, greatly impacting routine diagnoses and treatments of some chronic diseases, including Parkinson's disease (PD). In fact, the quality of life of patients with PD was seriously affected due to limited activity, inconvenience of acquiring prescriptions in hospitals, and concerns about COVID-19 infection. Of course, the difficulties of these patients would not last long. With the alleviation of COVID-19 epidemic in China, measures for epidemic prevention and control were gradually lifted from April 1, 2020. People could freely enter or leave public places when their body temperature was normal and their health code was green, rather than red or yellow.

During the COVID-19 pandemic, telemedicine has instantly transformed from a niche practice into the dominant means of providing care [2]. Doctors in China took great efforts to help their patients through telemedicine, which made telemedicine develop rapidly in this particular period [3].

To explore the influence of the COVID-19 epidemic on the quality of life of patients with PD and the importance of telemedicine for management of PD, we carried out an epidemiological survey involving 113 patients with PD from Xihu District, Hangzhou, Zhejiang.

## 2. Materials and Methods

### 2.1. General Materials

A total of 113 patients with PD were selected from Xihu District, Hangzhou, Zhejiang. The inclusion criteria for this study were as follows: (1) patients who met the Movement Disorder Society clinical diagnostic criteria of primary PD, (2) whose Hoehn Yahr stage was I–IV, and (3) who agreed to participate in the study. The exclusion criteria for the study were as follows: (1) patients with mental disorders or cognitive dysfunctions that seriously affected language expression, (2) those with secondary Parkinson's syndrome, (3) those with other inherited degenerative diseases or Parkinson's syndrome, and (4) those patients whose Hoehn Yahr stage was V.

### 2.2. Methods

During the epidemic prevention and control period (February 1 to March 31, 2020), the questionnaire survey method was adopted, following the principles of confidentiality and voluntary participation. The researchers conducted a detailed inquiry of the investigation content through several means of communication, such as face-to-face assessments, telephone visits, and social media software (WeChat or Tencent QQ). Then the patients filled out the questionnaires, including the “COVID-19 Questionnaire for PD Patients during the Period of Epidemic Prevention and Control” and the “39-item Parkinson's Disease Questionnaire” (PDQ-39). During the phase of gradually lifting epidemic prevention and control measures (April 1 to April 30, 2020), patients with PD could get help from neurologists through telephone calls, messages, or videos sent by social media software (WeChat or Tencent QQ). All patients enrolled in this study had follow-up appointments, and the PDQ-39 forms were completed again. All scale assessments were performed in the “open” phase of patient care. The questionnaires were collected at the beginning of May in 2020.

The study protocol was approved by the ethics committee at the local hospital. All enrolled patients or participants (caregivers) filled out written informed consent to be involved in this research.

### 2.3. Statistical Methods

SPSS version 20.0 statistical software (SPSS, Inc., Chicago, IL, USA) was used to process the experimental data. The measurement data were expressed using mean ± standard deviation, and the count data were tested using the *t*-test. *P* < 0.05 was statistically significant.

## 3. Results

### 3.1. General Information and Questionnaire Results of Patients

Among all the patients, 66 were male and 47 were female; the age range was 51–86 years, the average age was 69.5 ± 7.8, the course of the disease was 10–216 months, and the average duration was 72.6 ± 9.3 months. Each patient needed to fill in one “COVID-19 Questionnaire for PD Patients during the Period of Epidemic Prevention and Control” and two PDQ-39 questionnaires. In this study, a total of 113 copies of “COVID-19 Questionnaire for PD Patients during the Period of Epidemic Prevention and Control” and PDQ-39 forms were distributed, and 108 copies were effectively recovered. The recovery rate was 95.6%.  Table 1 shows the results of “COVID-19 Questionnaire for PD Patients during the Period of Epidemic Prevention and Control.”

### 3.2. Comparison of PDQ-39 Assessment Results between during and after Epidemic Prevention and Control

Comparison of all PDQ-39 domain outcomes between during and after epidemic prevention and control are shown in Table 2. Except for social support, the scores of mobility, activities of daily living, emotional well-being, stigma, cognition, communication, bodily discomfort, and total PDQ-39 scores during epidemic prevention and control were significantly higher than those after epidemic prevention and control (*P* < 0.001), indicating that the quality of life of patients with PD was significantly affected during the period of epidemic prevention and control.

### 3.3. Comparison of PDQ-39 Outcomes between during and after Epidemic Prevention and Control in Patients Who Could Easily Receive Doctor's Guidance and Those Who Found It Difficult

According to the results of item 4 in Table 1, patients who responded with a “yes” were classified as the “easy to get doctor's guidance” subgroup and those who responded with a “no” were classified as the “difficult to get doctor's guidance” subgroup. Subsequently, we compared the difference of PDQ-39 scores between during and after the epidemic prevention and control in the two subgroups separately. As shown in Table 3, only the outcomes of mobility, activities of daily living, emotional well-being, and total PDQ-39 scores during epidemic prevention and control were significantly higher (*P* < 0.05) than those after epidemic prevention and control in the “easy to get doctor's guidance” subgroup. Almost all domains, except for social support, showed significant differences in the “difficult to get doctor's guidance” subgroup (*P* < 0.001).

### 3.4. Comparison of PDQ-39 Outcomes between during and after Epidemic Prevention and Control in Patients Who Changed Routine Medication and Those Who did Not

According to the results of item 6 in Table 1, 79.6% (86) of these patients changed routine medication during the epidemic prevention and control while the remaining 20.4% (22) did not. The difference of PDQ-39 scores for during and after the epidemic prevention and control of the two parts was compared separately. As shown in Table 4, almost all domains, except for social support, showed significant differences (*P* < 0.001) in patients who changed routine medication, while only the outcomes of mobility, activities of daily living, emotional well-being, and total PDQ-39 scores showed significant difference (*P* < 0.05) in patients who did not change routine medication.

## 4. Discussion

The COVID-19 outbreak is now a worldwide topic. Currently, more and more people get infected and the death toll continues to rise. Without question, the COVID-19 pandemic has altered the way we practice neurology and our management of patients with movement disorders worldwide [4].

As a common chronic disease, PD needs long-term comprehensive management [5]. At present, drug therapy is the main treatment to control the symptoms of PD, which means that patients with this disease need regular medication to consistently control the symptoms. However, the COVID-19 outbreak and the consequent restrictions on travel have disrupted regular visits for patients with PD [6]. During the epidemic prevention and control period (February 1 to March 31, 2020), some hospitals even closed the Parkinson's clinics because of the pandemic, rendering it impossible for some Parkinson's patients to see a doctor on time, as well as causing worry about being infected and having irregular visits to the hospital [7]. However, there is currently insufficient evidence showing that PD by itself increases the risk of COVID-19 [8]. In accordance with China's medical security system, patients with PD could only purchase medication from hospitals with doctors' prescriptions. If they are unable to visit the hospital on time, they may be forced to decrease or even stop the anti-PD drugs.

From this investigation, it is clear that the COVID-19 outbreak is widely known among patients, and the main channel for them to obtain relevant information is through the community staff. This reason is because the community staff are responsible for the transmission of information about the pandemic to the residents and the management of personnel in and out of the community.

For patients with PD, the biggest problem during the epidemic prevention and control period has been that they cannot receive the doctor's guidance regularly. Most chose to go to the hospital with their family members or relatives, while some chose to contact their doctors using social media software (WeChat or Tencent QQ). Nevertheless, not all patients possess the ability to use social media on smartphones. Thus, a small number of patients have needed to visit the Parkinson's clinic by themselves. This necessity entails a high risk of being infected. However, on the positive side, of all the 108 patients surveyed, no one was definitely diagnosed with COVID-19. This is probably because Hangzhou was not a seriously affected area.

Patients with PD could develop different degrees of cognitive dysfunction [9], which may affect their understanding of the epidemic management program, resulting in anxiety, depression, or other emotional disorders. In addition, 79.6% of the patients did not receive their doctor's guidance in time and adjusted the drug dosage by themselves for the reasons of either shortage of the drugs or aggravation of symptoms. Most of the patients believed that their symptoms were aggravated. Among these, anxiety and depression were the main symptoms. The aggravation of rigidity and tardiness were also obvious. Some patients even suffered movement complications, such as dyskinesia and symptom fluctuation, which may be related to unreasonable drug adjustment.

As shown in the results, except for social support, the scores of mobility, activities of daily living, emotional well-being, stigma, cognition, communication, bodily discomfort, and total PDQ-39 scores during epidemic prevention and control were significantly higher than those after epidemic prevention and control, indicating that the quality of life of patients with PD was significantly affected during the period of epidemic prevention and control. The reason why the social support scores did not change significantly may be due to the family members of patients having more time to accompany them due to lockdown during the epidemic.

The results shown in Table 3 revealed that the quality of life of patients who had difficulty getting their doctor's guidance was more susceptible during the period of epidemic prevention and control. In addition, results shown in Table 4 indicated that the quality of life of patients who changed their routine medication due to lockdown was even worse.

The COVID-19 epidemic and corresponding travel restriction orders have also changed the way neurologists work. The global lockdown has forced some neurologists to practice from their homes and find new ways to manage neurological patients remotely [10]. In China, the daily workload of doctors had been reduced due to lockdown, which may have given them more time to improve the management of patients with PD through telemedicine. Doctors tried hard to guide the adjustment of therapeutic regimen of their patients through telephone or social media software (WeChat or QQ).

Fortunately, with the end of the epidemic prevention and control, patients' regular medical treatment was restored, and their quality of life improved rapidly. People's lives have gradually returned to normal, but telemedicine has been retained because the experience during the epidemic has proved that it is a quite effective and an efficient means to manage chronic diseases including PD [11]. Even after the COVID-19 emergency, telemedicine will be essential to streamline outpatient visits, while at the same time limiting costs [12].

## 5. Conclusions

This investigation shows that the lockdown during the epidemic will directly cause the aggravation of patients' symptoms and the decline in their quality of life. The quality of life of patients who had difficulty getting their doctor's guidance or those who changed their routine medication due to lockdown was even worse. Telemedicine is a quite effective and efficient means to manage chronic diseases including PD. It is suggested using social media (such as WeChat or Tencent QQ) for regular interactions and follow-up appointments if effective for patients with inconvenient medical treatment during the COVID-19 pandemic.

## Figures and Tables

**Table 1:** COVID-19 Questionnaire for PD Patients during the Period of Epidemic Prevention and Control.

Questions	Patients
1. Do you know about the COVID-19 pandemic?	
Yes	100% (108)
No	0% (0)

2. How did you find out about the COVID-19 pandemic?	
Television	70.4% (76)
Newspapers	29.6% (32)
Smart phone or other social media	50.0% (54)
Family members	72.2% (78)
The community staff	100% (108)
Doctors	20.4% (22)

3. Which is the biggest problem you are facing due to the COVID-19 pandemic?	
Unable to consult a doctor	60.2% (65)
Unable to procure supply of medication due to lockdown	24.1% (26)
Unable to go for walks due to lockdown	15.7% (17)

4. Could you get access to your doctor regularly?	
Yes	30.6% (33)
No	69.4% (75)

5. What are the main ways for you to get advice from your doctor during the period of epidemic control? (choose one or two)	
Smart phone apps or other social media	50.0% (54)
Family members or caregivers visit the Parkinson's clinic instead of patients	69.4% (75)
Go to the PD clinic directly	19.4% (21)

6. Have you adjusted the daily medication routine due to lockdown?	
Yes	79.6% (86)
No	20.4% (22)

7. If 6 is a “yes,” why?	
Insufficient reserve of drugs for PD	39.8% (43)
Worsening of symptoms	39.8% (43)

8. Have you experienced any new/worsening of symptoms following the onset of the COVID-19 pandemic?	
Yes	79.6% (86)
No	20.4% (22)

9. If 8 is a “yes,” then in which aspects?	
Increased tremor	39.8% (43)
Increased stiffness	60.2% (65)
Increased slowness	60.2% (65)
Newly appearing or worsening of dyskinesia	39.8% (43)
Newly appearing or worsening of fluctuation of symptoms	40.7% (44)
Excessive fatigue	29.6% (32)
Feeling/appear stressed or anxious	79.6% (86)
Feeling/appear depressed	50.0% (54)
Sleep disorders	39.8% (43)
Reduced appetite	10.2% (11)
Increased aches and pains (including any painful cramp or muscle spasm)	29.6% (32)

**Table 2:** Comparison of all PDQ-39 domain outcomes of 108 patients between during and after epidemic prevention and control.

Variables	During epidemic prevention and control	After epidemic prevention and control	*t* value	Sig
Mobility	20.42 ± 3.37	15.84 ± 3.37	16.961	*P* < 0.001
ADL	12.73 ± 2.36	10.41 ± 2.61	9.174	*P* < 0.001
Emotional well-being	12.51 ± 1.85	9.93 ± 2.42	11.691	*P* < 0.001
Stigma	8.60 ± 1.84	7.36 ± 2.01	6.955	*P* < 0.001
Social support	6.32 ± 1.47	6.06 ± 1.84	1.915	0.058
Cognition	8.39 ± 1.98	6.92 ± 2.07	7.714	*P* < 0.001
Communication	5.95 ± 1.55	5.08 ± 1.76	4.480	*P* < 0.001
Bodily discomfort	6.36 ± 1.68	4.38 ± 1.69	10.345	*P* < 0.001
Total score	81.29 ± 9.09	65.94 ± 8.70	20.363	*P* < 0.001

**Table 3:** Comparison of PDQ-39 outcomes between during and after epidemic prevention and control in patients who could easily receive doctor's guidance and those who found it difficult.

Variables		During epidemic prevention and control	After epidemic prevention and control	*t* value	Sig
“Easy to get doctor's guidance” subgroup (n = 33)	Mobility	19.33 ± 3.24	16.58 ± 3.70	6.273	*P* < 0.001
	ADL	11.88 ± 2.36	10.82 ± 3.19	2.726	0.010
	Emotional well-being	11.91 ± 1.86	10.73 ± 2.39	3.750	0.001
	Stigma	7.85 ± 1.72	7.30 ± 2.04	1.578	0.124
	Social support	5.55 ± 1.50	5.39 ± 1.78	0.645	0.523
	Cognition	7.73 ± 1.94	7.42 ± 2.05	0.796	0.432
	Communication	5.48 ± 1.40	5.55 ± 1.77	−0.201	0.842
	Bodily discomfort	5.88 ± 1.58	5.61 ± 1.80	0.851	0.401
	Total score	75.61 ± 9.08	69.39 ± 9.11	10.362	*P* < 0.001

“Difficult to get doctor's guidance” subgroup (n = 75)	Mobility	20.89 ± 3.34	15.67 ± 3.30	16.832	*P* < 0.001
	ADL	13.11 ± 2.28	10.23 ± 2.31	9.539	*P* < 0.001
	Emotional well-being	12.77 ± 1.79	9.57 ± 2.36	12.461	*P* < 0.001
	Stigma	8.93 ± 1.80	7.24 ± 1.97	9.153	*P* < 0.001
	Social support	6.67 ± 1.33	6.35 ± 1.80	1.838	0.070
	Cognition	8.68 ± 1.93	6.69 ± 2.05	10.388	*P* < 0.001
	Communication	6.16 ± 1.58	4.88 ± 1.72	5.514	*P* < 0.001
	Bodily discomfort	6.57 ± 1.69	3.84 ± 1.33	15.320	*P* < 0.001
	Total score	83.79 ± 7.94	64.47 ± 8.14	29.958	*P* < 0.001

**Table 4:** Comparison of PDQ-39 outcomes between during and after epidemic prevention and control in patients who changed routine medication and those who did not.

Variables		During epidemic prevention and control	After epidemic prevention and control	*t* value	Sig
Patients who changed routine medication (n = 86)	Mobility	20.80 ± 3.41	15.74 ± 3.37	17.330	*P* < 0.001
	ADL	13.03 ± 2.29	10.41 ± 2.44	9.172	*P* < 0.001
	Emotional well-being	12.80 ± 1.79	9.84 ± 2.44	12.270	*P* < 0.001
	Stigma	8.81 ± 1.85	7.26 ± 1.98	8.724	*P* < 0.001
	Social support	6.48 ± 1.45	6.20 ± 1.80	1.716	0.090
	Cognition	8.62 ± 1.98	6.85 ± 2.06	9.107	*P* < 0.001
	Communication	6.10 ± 1.62	4.99 ± 1.74	5.095	*P* < 0.001
	Bodily discomfort	6.56 ± 1.65	4.13 ± 1.58	13.061	*P* < 0.001
	Total score	83.21 ± 8.43	65.41 ± 8.77	24.602	*P* < 0.001

Patients who did not change routine medication (n = 22)	Mobility	18.91 ± 2.81	16.73 ± 3.67	4.446	*P* < 0.001
	ADL	11.55 ± 2.30	10.41 ± 3.25	2.411	0.025
	Emotional well-being	11.36 ± 1.62	10.27 ± 2.33	2.693	0.014
	Stigma	7.77 ± 1.57	7.27 ± 2.00	1.111	0.279
	Social support	5.73 ± 1.45	5.50 ± 1.92	0.839	0.411
	Cognition	7.50 ± 1.74	7.18 ± 2.13	0.656	0.519
	Communication	5.36 ± 1.09	5.45 ± 1.82	−0.253	0.803
	Bodily discomfort	5.59 ± 1.59	5.36 ± 1.79	0.527	0.604
	Total score	73.77 ± 7.68	68.18 ± 8.30	8.828	*P* < 0.001

## Data Availability

The data are available from the corresponding author upon reasonable request.
